# The sound of trustworthiness: Acoustic-based modulation of perceived voice personality

**DOI:** 10.1371/journal.pone.0185651

**Published:** 2017-10-12

**Authors:** Pascal Belin, Bibi Boehme, Phil McAleer

**Affiliations:** 1 La Timone Neuroscience Institute, Mixed Research Unit 7289 Centre National de la Recherche Scientifique and Aix-Marseille University, Marseille, France; 2 School of Psychology, Institute of Medicine Veterinary and Life Sciences, University of Glasgow, Glasgow, United Kingdom; 3 Département de Psychologie, Université de Montréal, Montréal, Québec, Canada; Tokai University, JAPAN

## Abstract

When we hear a new voice we automatically form a "first impression" of the voice owner's personality; a single word is sufficient to yield ratings highly consistent across listeners. Past studies have shown correlations between personality ratings and acoustical parameters of voice, suggesting a potential acoustical basis for voice personality impressions, but its nature and extent remain unclear. Here we used data-driven voice computational modelling to investigate the link between acoustics and perceived trustworthiness in the single word "hello". Two prototypical voice stimuli were generated based on the acoustical features of voices rated low or high in perceived trustworthiness, respectively, as well as a continuum of stimuli inter- and extrapolated between these two prototypes. Five hundred listeners provided trustworthiness ratings on the stimuli via an online interface. We observed an extremely tight relationship between trustworthiness ratings and position along the trustworthiness continuum (r = 0.99). Not only were trustworthiness ratings higher for the high- than the low-prototypes, but the difference could be modulated quasi-linearly by reducing or exaggerating the acoustical difference between the prototypes, resulting in a strong caricaturing effect. The f0 trajectory, or intonation, appeared a parameter of particular relevance: hellos rated high in trustworthiness were characterized by a high starting f0 then a marked decrease at mid-utterance to finish on a strong rise. These results demonstrate a strong acoustical basis for voice personality impressions, opening the door to multiple potential applications.

## Introduction

When we see a new face or hear a new voice, we automatically form a first impression of the person’s personality, inferring traits such as trustworthiness, competence, attractiveness, etc. [1–5]. These personality impressions may not be accurate but they are robust, showing high inter-rater agreement [1, 3, 4, 6]; they also form very rapidly as sub-second exposure to face or voice is sufficient to yield consistent judgments [4, 7].

For both faces and voices, first impressions are well summarized by a 2-dimensional « social space » with axes mapping onto perceived trustworthiness and dominance, respectively [4, 6]. These two dimensions are thought to reflect evolutionary ancient mechanisms, developed under selective pressures of living in large social groups, to rapidly assess the dispositions (trustworthiness) and ability to act on these dispositions (dominance) of an unfamiliar conspecific individual [8, 9].

For faces, data-driven computational modelling has demonstrated a very tight relationship between perceived personality impressions and specific facial features [1, 10]. The generation of model faces at different positions of the “social face space” shows that the main dimensions of trustworthiness and dominance map onto very different sets of facial features, such as jaw shape vs. eyebrow shape.

For voices, significant correlations between personality ratings and acoustical parameters such as average fundamental frequency (f0) have been observed [2, 4–6, 11, 12] suggesting an acoustical basis for voice personality judgments by which principled changes in voice acoustics could potentially map onto desired changes in perceived personality. But until now the correlational approach has fallen short of precisely characterizing this acoustical basis. *How exactly should one say “hello” to be perceived as e*.*g*., *trustworthy by new listeners*?

Here we used voice computational modelling to investigate more closely the acoustical basis for perceived voice personality. We focused on perceived trustworthiness because due to its evolutionary link to cooperation and survival, trust is a central aspect of human communication, the foundation for functioning relationships, interpersonal interaction, effective collaboration [13] as well as the cooperation and mutual adaptation needed to surmount problems [14, 15]. Ratings along that trait were found in a previous study to correlate most strongly with the first principal component explaining more than half the variance in overall voice personality ratings [4], indicating the foremost role of perceived trustworthiness in overall voice personality impressions.

We first asked whether voices modeled as the acoustical average of several low- and high-trustworthy voices would also be rated with low and high perceived trustworthiness—potential evidence of a mapping between acoustical and social voice spaces. We further explored the possible parametric nature of the acoustics-personality link by collecting trustworthiness ratings for voice stimuli generated in voice space along the direction defined by the low- and high-trustworthiness prototypes, including caricatures of one relative to the other.

**Fig 1:**
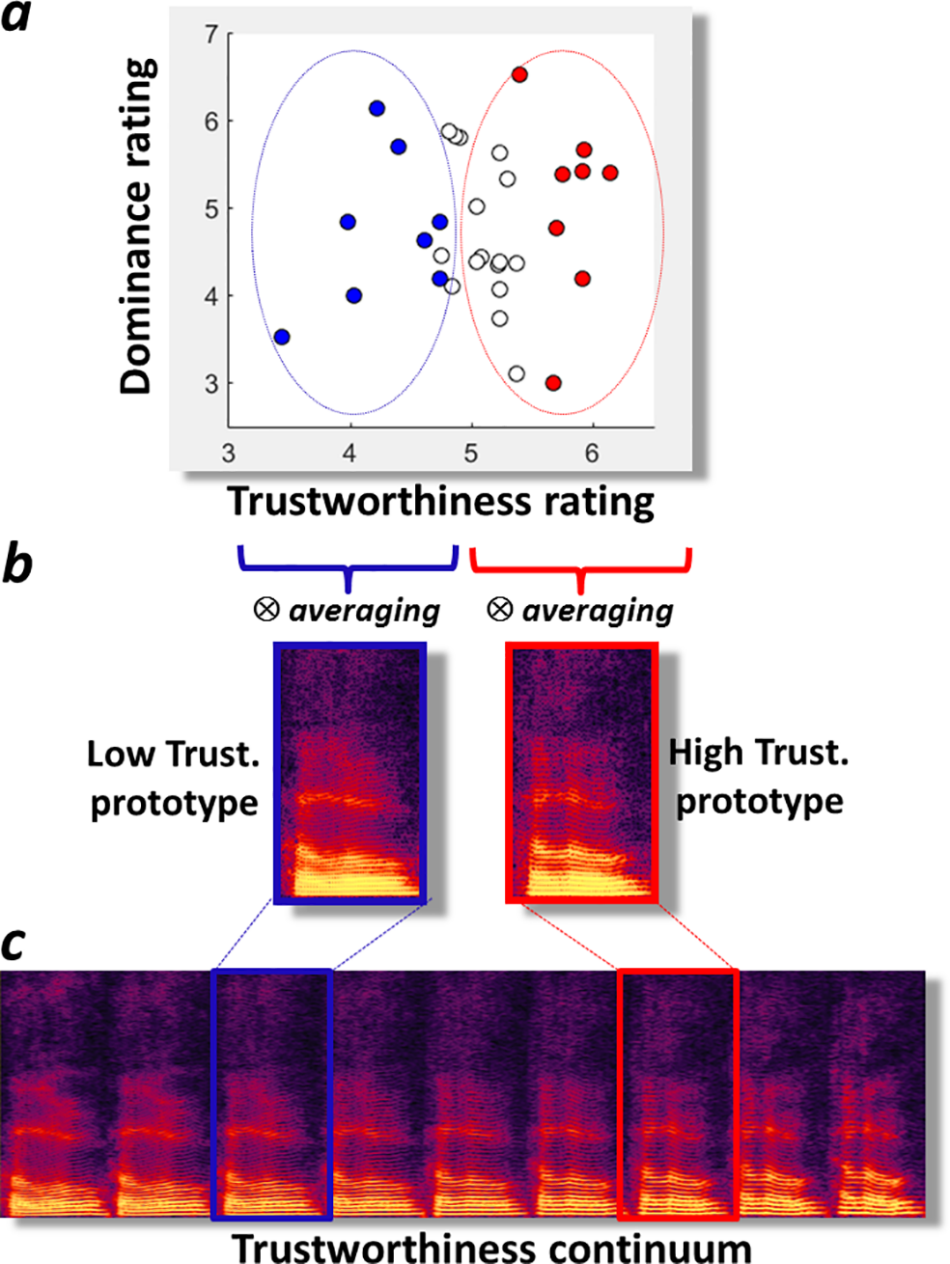
Generation of the voice trustworthiness continuum. **a.** Thirty-two male voices saying “hello” are represented as points in a 2D “social voice space” with axes mapping onto their perceived Trustworthiness and Dominance, respectively (cf. [4]). Coloured dots indicate voices within the bottom (blue dots) and top (red dots) 25% for Trustworthiness ratings. **b.** A Low- and a High-Trustworthiness prototype were generated by averaging via morphing the bottom and top 25% rated voices, respectively. **c.** A 9-stimulus “Trustworthiness continuum “was generated via morphing in 8 acoustically equal steps using the two prototypes in intermediate positions and extrapolating to low- and high- Trustworthiness caricatures at extreme positions.

Thirty-two male voices saying the word “hello” were ranked by their average trustworthiness ratings collected as part of a previous study [4] (Fig 1A). A low- and a high-trustworthiness prototypical model voice stimuli were generated via high-quality analysis/resynthesis [16] based on the average acoustical characteristics of the eight voices (25%) rated lowest and highest on perceived trustworthiness, respectively (Fig 1B; S1 Fig; cf. Methods). This resulted in two natural-sounding “hellos” (cf. audio files in Supporting Information) with perceptually different voice qualities and which we hypothesized would yield different trustworthiness ratings.

Next we generated via morphing a 9-stimulus “Trustworthiness continuum” in acoustically equal steps that contained the low- and high-trustworthiness prototypes at intermediate positions (stimuli S3 and S7) and that inter- and extrapolated their acoustical difference resulting at the extreme in low- and high-trustworthiness caricatures (stimuli S1 and S9; Fig 1C). To provide a set of control stimuli generated similarly but unrelated to trustworthiness we generated a second 9-stimulus “Control continuum” based on a random selection of a set of 32 female voices (S2 Fig; audio files in Supporting Information).

Five hundred listeners (146 males, 354 females; aged 19–65 years old, median age = 24) each provided 18 trustworthiness ratings using a visual analogue scale via an online interface, 9 for the morphed voice stimuli of the Trustworthiness continuum and 9 for those of the control continuum (stimuli presented in random orders in counterbalanced blocks; cf. Methods). Ratings were z-scored within participant prior to analysis to account for inter-individual variation in rating scale use and because we were primarily interested in rating variations within the morphed continua rather than in their absolute values.

## Results and discussion

**Fig 2:**
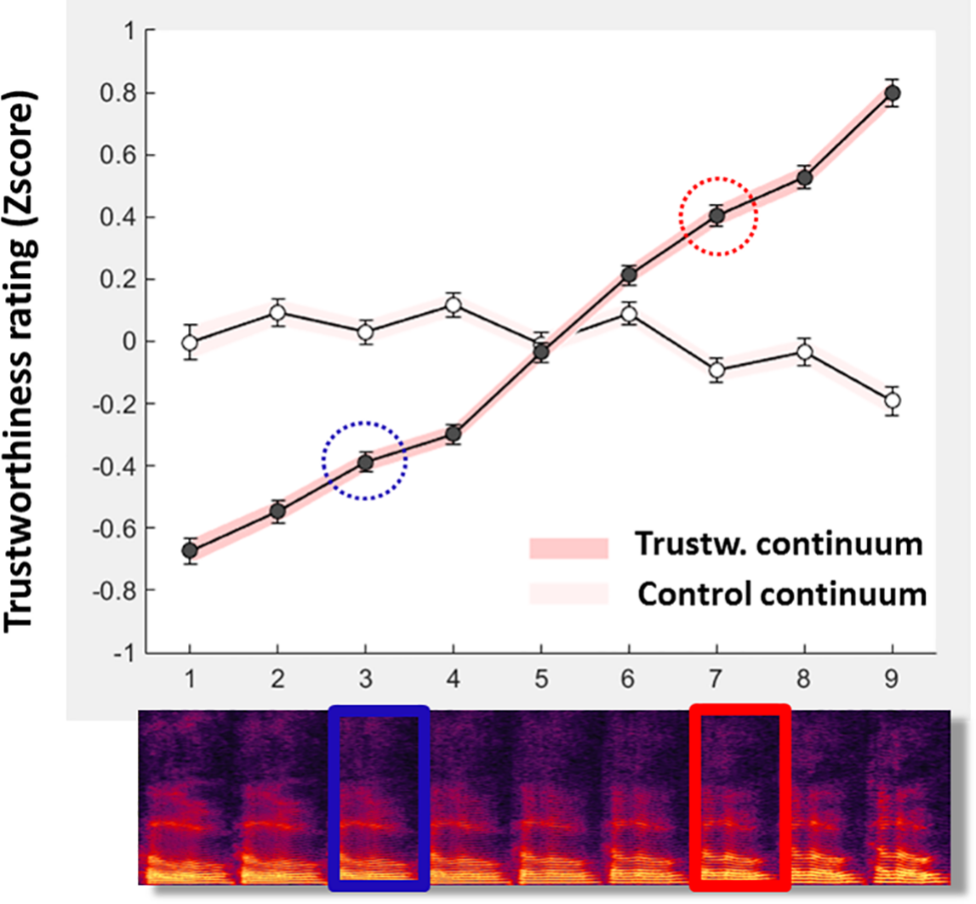
Acoustic-based modulation of perceived voice trustworthiness. Filled circles indicate group-average Trustworthiness rating z-scores for each of the 9 stimuli of the Trustworthiness continuum (spectrograms at bottom) from low-Trustworthiness caricature (S1) to low- Trustworthiness prototype (S3; blue rectangle), to the average of the two prototypes (S5), to the high-Trustworthiness prototype (S7; red rectangle) to the high- Trustworthiness caricature (S9). Note the quasi-linear modulation of Trustworthiness ratings along the Trustworthiness continuum. Open circles show average T-rating z-scores for the control continuum. Error bars and shaded areas indicate mean ± s.e.m.

We observed a compelling relationship between average trustworthiness rating z-scores and position along the Trustworthiness, but not the Control, continuum (Fig 2). Ratings for the low- and high- trustworthiness prototypes (stimuli S3 and S7) differed highly significantly with lower average ratings for S3 than S7 (S3: z = -0.39; S7: z = 0.40; paired t-test: t(499) = 14.2, p = 1.38e-38) while no such difference was apparent for control stimuli (S3: z = 0.03; S7:z = -0.09; t(499) = 2.08, p = 0.038 n.s. after Bonferroni correction).

We also observed a strong caricaturing effect, as exaggerating acoustical differences between S3 and S7 yielded higher average trustworthiness ratings for S9 than S7 (S7: z = 0.40; S9; z = 0.80; t(499) = 7.40, p = 5.93e-13), and lower average trustworthiness ratings for S1 than for S3 (S1: z = -0.67; S3: z = -0.38; t(499) = 6.06; p = 2.62e-9). In fact, the average ratings followed an extremely tight relation with acoustical differences over the whole range of the continuum generated resulting in a correlation of r = 0.99 (p = 5.03e-8) between position along the acoustical continuum and average ratings. Again no such effect was observed for control stimuli on either end of the continuum (S1: z = 0; S3: z = 0.03; t(499) = -0.49, p = 0.62 n.s.; S7: z = -0.09; S9: z = -0.19; t(499) = 1.63, p = 0.10 n.s.). Importantly these effects were highly similar for male and female listeners (S3 Fig) suggesting the involvement of gender-independent mechanisms.

These results demonstrate a strong acoustical basis for perceived voice personality—an extremely tight mapping between acoustical and social voice spaces. Not only does averaging voices from different individuals with low or high ratings trustworthiness ratings results in the predicted rating differences, but these personality impressions can be parametrically modulated, with unsuspected accuracy, by acoustical changes and considerably increased (or decreased) by caricaturing.

Furthermore the results allow *listening* to the prototypes and caricatures, permitting direct access to the listeners’ underlying representations of voice trustworthiness in a way that correlations between traits and acoustical measures could not allow. The comparison of the prototypes and caricatures’ acoustical measures not only gives insight on which acoustical parameters are relevant, it directly shows *how* to modulate these parameters in order to achieve the desired increase in perceived trustworthiness.

**Fig 3:**
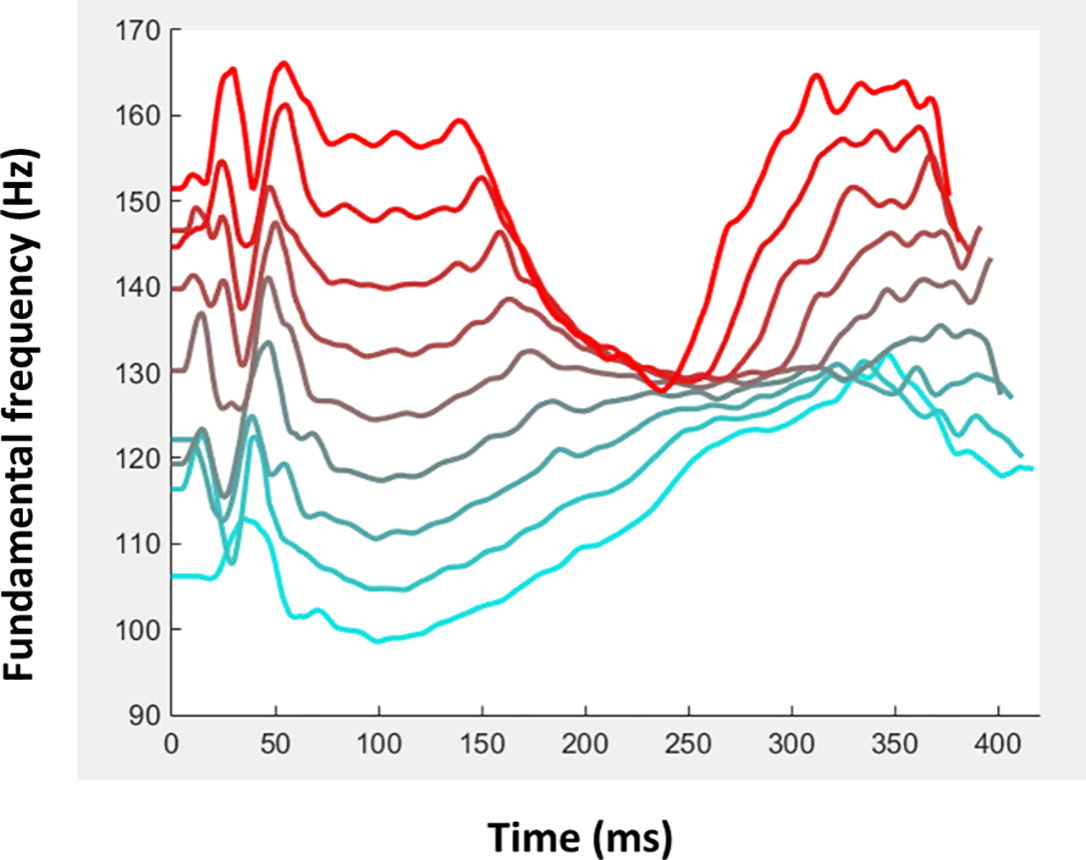
Intonation and perceived trustworthiness. The f0 contours of the 9 stimuli of the Trustworthiness continuum (from light blue for stimulus S1 to red for stimulus S9) are shown illustrating a marked change in intonation as perceived trustworthiness increases. While low trustworthiness stimuli have a flat or slightly rising f0 contour, high-trustworthiness stimuli are characterized by a marked f0 drop at the end of the first syllable to finish the second syllable on a markedly rising f0.

Thus, past studies have hinted at a relation between f0 variation in voice and perceived personality traits such as attractiveness [5, 11, 17] or trustworthiness [4] but they could not determine the exact f0 contour that would drive this percept. What intonation should one use when saying “hello” to be perceived as trustworthy? Fig 3 provides important elements of answer to that question by illustrating f0 contour for the 9 stimuli of the Trustworthiness continuum. F0 at mid-duration is roughly similar for all stimuli but a clear difference is apparent for starting and ending f0: while the low-trustworthiness stimuli started with a relatively low f0 remaining flat or slightly increasing over the course of the syllables, the high-trustworthiness stimuli started with a much higher f0 at first syllable then decreased markedly to reach the average f0 at mid utterance and finish on a strongly rising intonation (cf. High trustworthiness caricature, Fig 3, audio file in Supporting Information). While f0 contour is but one of the many only acoustical parameters varying between stimuli (S1 Table) this particular intonation likely plays a central role on yielding the observed change in perceived personality impressions. Further work should weigh the respective importance of the different parameters by selectively including them or not in the prototypical models, for instance by generating an acoustic continuum that only manipulates f0 between the different stimuli, all other parameters being kept constant.

The results could potentially generalize to other personality traits, providing unprecedented insight into the voice acoustics that drive the perception of different traits and opening numerous possibilities for research. They also open the door to practical applications where desired personality impressions need to be achieved. Indeed, there are many situations in life where voice is the only cue available to form a new person’s first impression and where that first impression strongly influences future interactions. For example, telephone interviewers face the task of establishing trust a creating a positive impression with only vocal cues. Since the great majority of refusals occur within the opening phrases [18], a positive attitude towards both the subject and interviewer could be induced early on via acoustical modulation, resulting in increased response rates. Furthermore, speech synthesis and computer-generated voices foe e.g. social robots could be addressed as a promising area for applications in the customer service sector.

Some limitations of the study should be mentioned: They were only obtained on the word “hello”, which is justified as this word is often associated with first encounters, yet it is unclear how they would generalize other words or longer utterances. Moreover, the 8-voices averages are probably but a crude approximation of the listeners’ internal representations of trustworthy and untrustworthy voices; results could likely be further enhanced by using high-level reverse correlation for a more precise estimation of the listeners’ internal templates [19, 20]. Our choice of female voices for the control condition and male voice for the experimental condition was entirely arbitrary; it will be important in future studies to examine the potential role of factors such as gender, but also age, ethnicity, accent, verbal content, etc. on the acoustical basis for voice personality impressions.

## Methods

### Participants

500 participants (146 males, 354 females; aged 19–65 years old, median age = 24) took part in the voice rating experiment. Participants were recruited via email and social networking sites and directed to the web address of the experiment. Experimental procedures were approved by the University of Glasgow ethics committee and the study was conducted in accordance with the ethical standards laid down in the Declaration of Helsinki. Participants gave informed consent prior to the experiment, via reading a series of statements regarding freedom to withdraw, anonymity of responses and secured storage of data. As the experiment was carried out online, they confirmed that they had read and agreed to these statements by registering on the website. Participants were not permitted to take part without providing consent.

### Stimuli

Voice stimuli were modelled and generated using STRAIGHT [16] in Matlab (Mathworks, Inc., Natick, USA). STRAIGHT performs an instantaneous pitch-adaptive spectral smoothing in each stimulus for separation of contributions to the voice signal arising from the glottal source vs. supra-laryngeal filtering. A natural voice stimulus is decomposed by STRAIGHT into five parameters: f0, frequency, time, spectro-temporal density and aperiodicity that can be manipulated and combined across stimuli independently of one another, then synthesized into a novel voice stimulus. Time-frequency landmarks to be put in correspondence across voices during morphing were manually identified in each stimulus and corresponded to the frequencies of the first 3 formants at temporal landmarks corresponding to onset and offset of phonation or formant transitions (S1 Fig).

The low- and high-trustworthiness prototypes were generated by averaging each voice parameter across the eight voices having obtained lowest or highest average trustworthiness ratings, respectively, in a previous study [4]. These two prototypes were then used to generate a 9-stimulus acoustic continuum in eight steps of equal acoustical distance (25% of the difference between the two prototypes) by inter- and extrapolation between the low-trustworthiness prototype in position S3 and the high-trustworthiness prototype in position S7. Thus the respective weights of S3 and S7 for the 9 stimuli of the continuum were: S1: 150%/-50%; S2: 125%:-25%;S3: 100%/0%; S4: 75%/25%; S5: 50%/50%; S6:25%/75%; S7: 0%/100%; S8: -25%125%; S9: -50%/150%. A second continuum (Control Continuum) was generated using the exact similar approach using the female voice recordings and a random selection of the 16 underlying voices (Audio files in Supporting Information). Acoustical measures of the stimuli were obtained using VoiceSauce [21] and are shown in S1 Table. Spectrograms of the 9 stimuli of the Trustworthiness continuum are shown in Fig 1C, and their f0 contours in Fig 3. Spectrograms of stimuli from the Control continuum are shown in S2 Fig.

### Procedures

The experiment was carried out online on Glasgow University’ Institute of Neuroscience and Psychology experiments website (http://experiments.psy.gla.ac.uk/). Participants performed two blocks of ratings (order counterbalanced across subjects): they were either presented with male voice block first, followed by control voices (female) or vice versa, and asked to rate each voice on a continuum of trustworthiness represented by a visual analogue scale ranging from “very untrustworthy” to “very trustworthy”. At the start of each block, participants were asked, “For each voice, please rate how trustworthy it sounds, that is, how much you would be ready to trust that person.” No contextual information for the experiment was given, akin to a “no acquaintance” scenario. All voices were heard once per block with presentation order randomised in both blocks to avoid possible order effects. Participants were given the opportunity to take an untimed break between the two blocks.

Rating data are available as supporting information S1 Data.

### Analysis

Raw trustworthiness ratings (range 0–500) were converted to individual z-scores based on the mean and standard deviation of the 18 ratings provided by each participant. For both the Trustworthiness and Control continua, the distributions of individual z-scores were compared between the S3 and S7 stimuli via paired t-tests. A possible caricaturing effect was also examined via paired t-tests comparing S1 to S3 and S9 to S7 in each continuum. Bonferronni correction was applied to account for the 3 comparisons per continuum.

## Supporting information

S1 Audio File Trustworthiness_continuum.wav: The 9 stimuli played in succession from S1 to S9 (windows PCM format, 16-bit, mono, 16 kHz sampling rate). (WAV)

S2 Audio File Control_continuum.wav: The 9 stimuli of the control continuum played in succession from S1 to S9. (WAV)

S1 Data A.zip archive containing raw ratings from the 500 participants for the control and the trustworthiness continua. (ZIP)

S1 Fig Voice morphing. Panels show spectrograms of the word “hello” spoken by three male speakers. Dotted lines and black circles indicate time and frequency landmarks, respectively, put in correspondence across speakers during morphing.
(TIF)

S2 Fig Spectrograms of the 9-stimulus control continuum. (TIF)

S3 Fig Ratings by listener gender. (TIF)

S1 Table Acoustical measures. (PDF)
