# Correction: The sound of trustworthiness: Acoustic-based modulation of perceived voice personality

**DOI:** 10.1371/journal.pone.0211282

**Published:** 2019-01-17

**Authors:** Pascal Belin, Bibi Boehme, Phil McAleer

## Notice of republication

An incorrect version of S1 Data was published in error. This article was republished on January 10, 2019 to correct for this error. Please download this article again to view the correct version.
