# Supplementary figures and images for: The sound of trustworthiness: Acoustic-based modulation of perceived voice personality
Source: PLoS One. 2017 Oct 12;12(10):e0185651. doi: 10.1371/journal.pone.0185651 (PMC5638233; doi:10.1371/journal.pone.0185651)

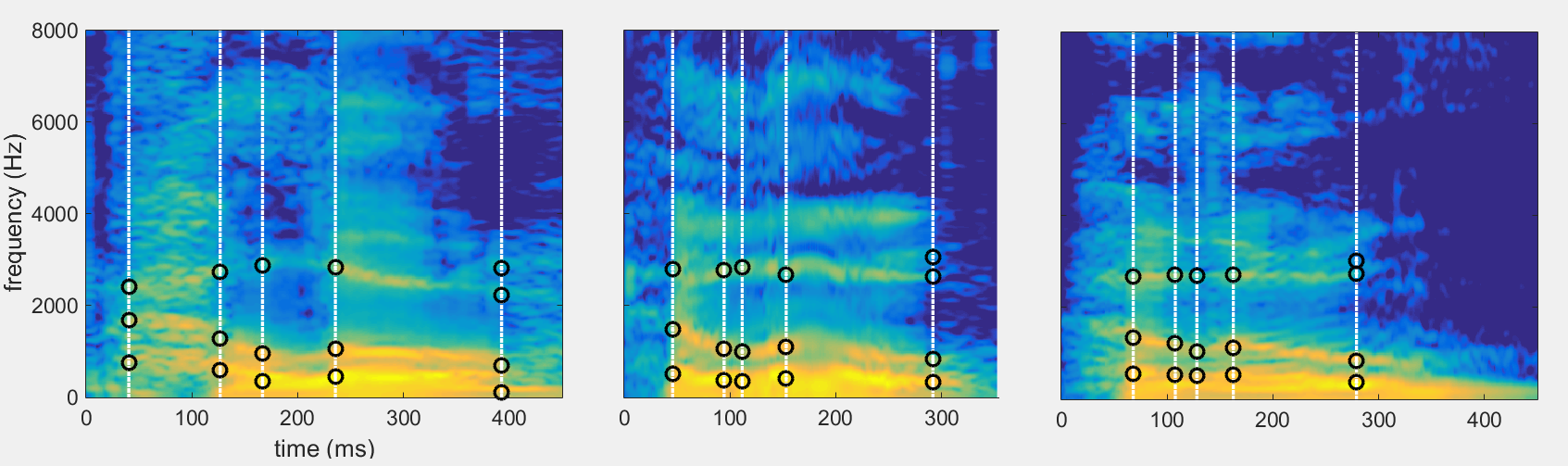

Supplement: S1 Fig — Panels show spectrograms of the word “hello” spoken by three male speakers. Dotted lines and black circles indicate time and frequency landmarks, respectively, put in correspondence across speakers during morphing. (TIF) [file pone.0185651.s004.tif]

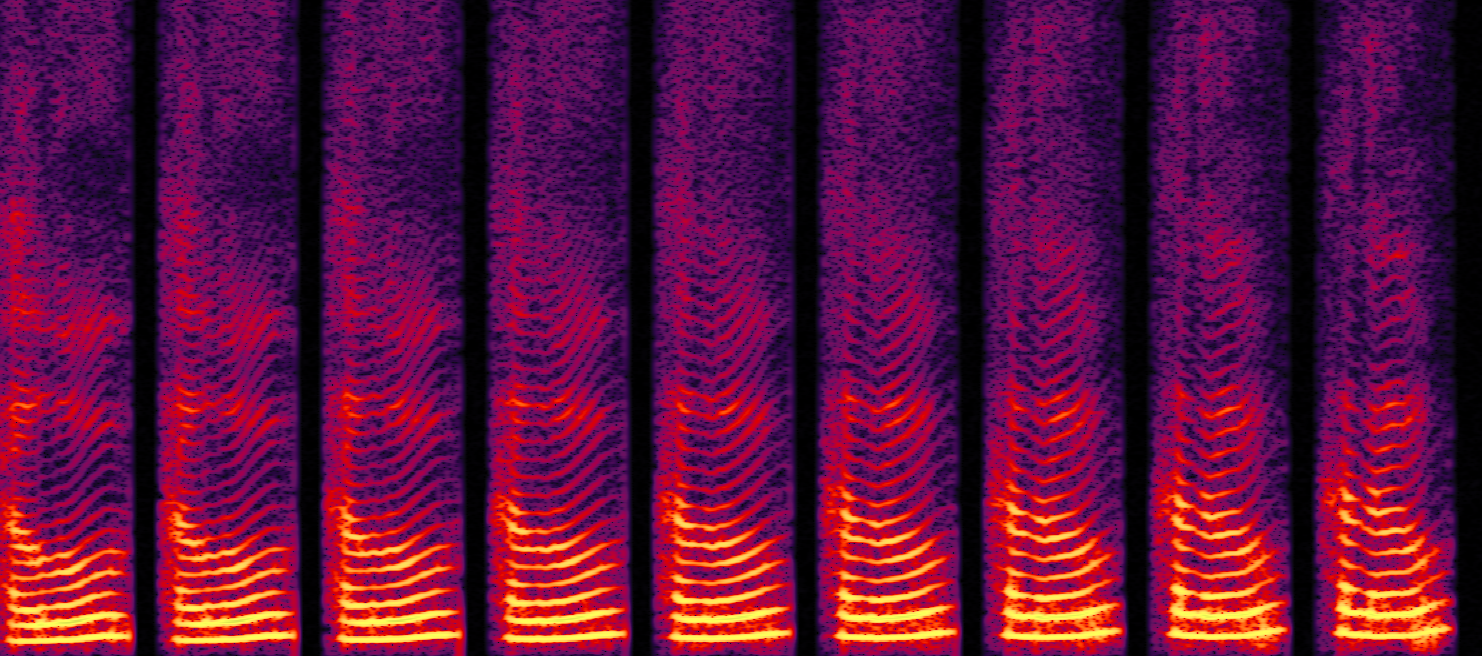

Supplement: S2 Fig — (TIF) [file pone.0185651.s005.tif]

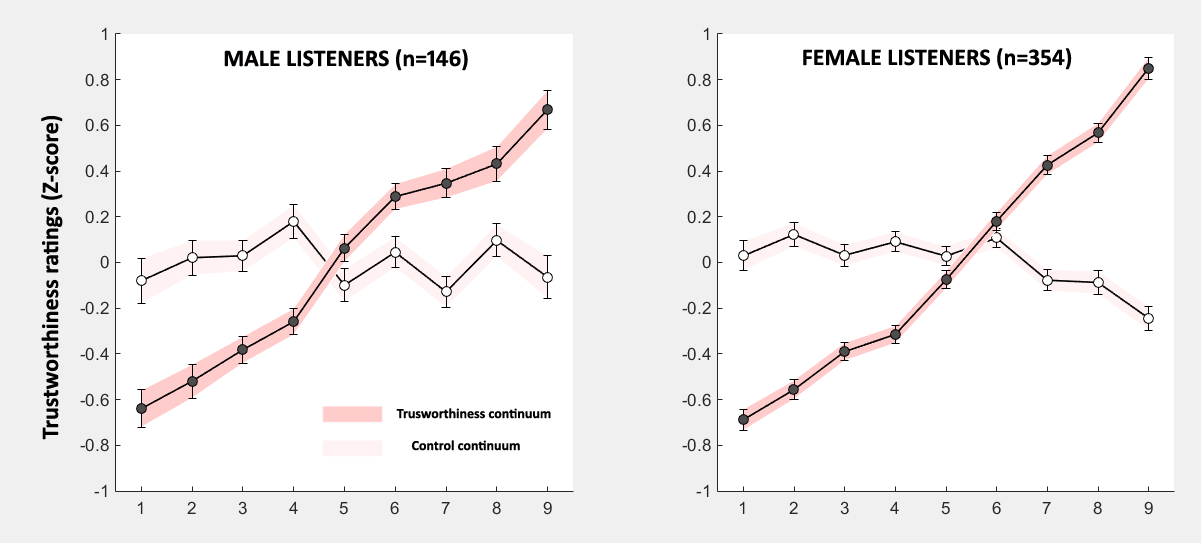

Supplement: S3 Fig — (TIF) [file pone.0185651.s006.tif]
