# Supplementary material for: The sound of trustworthiness: Acoustic-based modulation of perceived voice personality
Source: PLoS One. 2017 Oct 12;12(10):e0185651. doi: 10.1371/journal.pone.0185651 (PMC5638233; doi:10.1371/journal.pone.0185651)
Supplement: S1 Table — (PDF) [file pone.0185651.s007.pdf]

	Duration (ms)	avgf0 (Hz)	minf0 (Hz)	maxf0 (Hz)	stdf0 (Hz)	avgF1 (Hz)	avgF2 (Hz)	avgF3 (Hz)	avgF4 (Hz)	HNR (dB)	Energy (a.u.)
Control Continuum											
S1	343	242	205	300	31,2	542,9	1358	2810	4050	17,8	20,2
S2	355	241	206	314	31,6	524,7	1352	2836	4111	19,6	20,9
S3	367	235	190	309	33,0	516,5	1328	2836	4120	21,4	22,2
S4	379	235	185	322	37,5	504,3	1308	2845	4175	22,0	24,5
S5	391	235	185	327	42,8	499,3	1290	2870	4233	23,7	25,2
S6	403	235	192	328	48,1	488,2	1265	2887	4212	23,4	24,7
S7	415	235	182	335	54,0	492,7	1243	2915	4254	23,5	23,6
S8	427	236	164	344	60,0	483,0	1224	2935	4238	21,8	23,4
S9	439	236	153	352	66,5	481,1	1203	2985	4236	20,8	23,2
Trustworthiness Continuum											
S1	416	113	99	132	10,2	489,1	1179	2788	4016	11,1	37,5
S2	411	118	105	131	8,2	494,3	1132	2788	3896	13,8	32,7
S3	406	122	111	131	6,5	488,5	1169	2774	3934	16,1	28,0
S4	401	126	115	135	5,3	501,0	1217	2809	3988	17,1	25,7
S5	396	131	124	143	4,8	472,8	1178	2739	3945	16,0	25,1
S6	391	136	129	148	5,5	474,2	1253	2779	3992	13,9	21,8
S7	386	141	129	155	6,9	470,7	1226	2747	3967	11,3	24,3
S8	381	146	129	161	9,0	473,0	1277	2761	4031	10,9	23,7
S9	376	152	128	166	11,4	484,5	1403	2816	4118	9,9	21,1
